# Dimethyl Sulfoxide Inhibits Bile Acid Synthesis in Healthy Mice but Does Not Protect Mice from Bile-Acid-Induced Liver Damage

**DOI:** 10.3390/biology12081105

**Published:** 2023-08-09

**Authors:** Xi Chen, Huiqiao Li, Yu’e Liu, Jing Qi, Bingning Dong, Shixia Huang, Shangang Zhao, Yi Zhu

**Affiliations:** 1 USDA/ARS Children’s Nutrition Research Center, Department of Pediatrics, Baylor College of Medicine, Houston, TX 77030, USA; 2 Tongji University Cancer Center, Shanghai Tenth People’s Hospital of Tongji University, School of Medicine, Tongji University, Shanghai 200092, China; 3 Department of Emergency, The Third Xiangya Hospital of Central South University, Changsha 410013, China; qijing@csu.edu.cn; 4 Gastroenterology, Department of Medicine, Baylor College of Medicine, Houston, TX 77030, USA; 5 Department of Molecular and Cellular Biology, Baylor College of Medicine, Houston, TX 77030, USA; 6 Barshop Institute for Longevity and Aging Studies, Division of Endocrinology, Department of Medicine, University of Texas Health Science Center at San Antonio, San Antonio, TX 78229, USA

**Keywords:** dimethyl sulfoxide (DMSO), bile acid, liver damage, nonalcoholic steatohepatitis (NASH)

## Abstract

**Simple Summary:**

Bile acids are crucial in breaking down and absorbing fats. However, in excessive amounts, they can damage the liver. Our research investigated whether dimethyl sulfoxide (DMSO) could diminish bile acid production and subsequently protect the liver in mice. Our results showed that DMSO effectively reduced bile acid production in mouse primary hepatocytes and in vivo. Yet, DMSO failed to protect the liver when we evaluated its efficacy in two separate mouse models with liver damage induced or partially induced by excess bile acids. Notably, while DMSO decreases hepatic bile acid levels in healthy mice, the body appears to counterbalance this effect under disease conditions, resulting in persistent liver damage. These outcomes confirm that DMSO is not merely an inert solvent, but a biologically active agent. However, it falls short in treating liver diseases precipitated by elevated bile acid levels.

**Abstract:**

Bile acids serve a vital function in lipid digestion and absorption; however, their accumulation can precipitate liver damage. In our study, we probed the effects of dimethyl sulfoxide (DMSO) on bile acid synthesis and the ensuing liver damage in mice induced by bile acids. Our findings indicate that DMSO efficaciously curbs bile acid synthesis by inhibiting key enzymes involved in the biosynthetic pathway, both in cultured primary hepatocytes and in vivo. Contrarily, we observed that DMSO treatment did not confer protection against bile-acid-induced liver damage in two distinct mouse models: one induced by a 0.1% DDC diet, leading to bile duct obstruction, and another induced by a CDA-HFD, resulting in non-alcoholic steatohepatitis (NASH). Histopathological and biochemical analyses unveiled a comparable extent of liver injury and fibrosis levels in DMSO-treated mice, characterized by similar levels of increase in *Col1a1* and *Acta2* expression and equivalent total liver collagen levels. These results suggest that, while DMSO can promptly inhibit bile acid synthesis in healthy mice, compensatory mechanisms might rapidly override this effect, negating any protective impact against bile-acid-induced liver damage in mice. Through these findings, our study underscores the need to reconsider treating DMSO as a mere inert solvent and prompts further exploration to identify more effective therapeutic strategies for the prevention and treatment of bile-acid-associated liver diseases.

## 1. Introduction

The FDA classified dimethyl sulfoxide (DMSO) in the safest solvent category—the same class as ethanol—which has prompted it to be widely used in many biomedical fields, especially for cryopreservation and in vitro assays [1]. There is a common assumption that it is inert. In this study, we were interested in using DMSO to dissolve a compound for hepatocyte bile acid research. In a serendipitous discovery, we found that DMSO could dose-dependently inhibit several bile acid synthase genes, which led us to study whether DMSO inhibits bile acid metabolism in vivo and whether DMSO can be used as a treatment for bile-acid-induced liver damage.

It has been suggested that very few genes are affected by DMSO in human and rat hepatocytes below a toxic concentration (0.5% *v*/*v*) [2]. However, an increasing body of evidence suggests that this is not the case, and recent reports leveraging multi-omics show drastic changes in gene expression, microRNAs, and epigenetic landscape in 3D cardiac and hepatic microtissues induced by DMSO [1]. DMSO can also promote hepatocyte differentiation in vitro, probably through the upregulation of genes regulated by PXR and PPAR [3].

Bile acids are essential molecules synthesized in the liver from cholesterol and play a crucial role in the digestion and absorption of dietary fats and fat-soluble vitamins [4]. They also serve as signaling molecules, regulating various metabolic processes and liver functions [5,6,7]. Bile acid synthesis is a complex process involving multiple enzymes encoded by various genes. There are two major pathways for bile acid synthesis: the classical (or neutral) pathway and the alternative (or acidic) pathway.

The classical pathway, also known as the neutral pathway, is the primary route for bile acid synthesis, accounting for approximately 75–90% of total bile acid production in humans. Cholesterol 7 alpha-hydroxylase encoded by *CYP7A1* is the rate-limiting enzyme in the classical pathway, responsible for converting cholesterol into 7α-hydroxycholesterol [8]. Other enzymes in this pathway are encoded by genes *HSD3B7*, *CYP8B*, and *CYP27A1*. The alternative pathway, also known as the acidic pathway, serves as a minor route for bile acid synthesis, accounting for approximately 10–25% of total bile acid production in humans [9]. Sterol 27-hydroxylase, encoded by *CYP27A1*, is the rate-limiting enzyme in the alternative pathway, responsible for converting cholesterol into 27-hydroxycholesterol. Meanwhile, 25-hydroxycholesterol 7-alpha-hydroxylase, encoded by *CYP7B1*, converts 27-hydroxycholesterol into 3β,7α-dihydroxy-5-cholestenoic acid for the synthesis of chenodeoxycholic acid, one of the primary bile acids, in the alternative pathway. These are the primary genes and enzymes involved in bile acid synthesis, but several other enzymes and transporters also contribute to the process.

Biliary atresia is a rare and life-threatening congenital disorder affecting the liver and bile ducts in infants [10]. In this condition, the bile ducts that transport bile from the liver to the small intestine become inflamed, obstructed, or completely absent. Bile accumulates in the liver when it cannot flow through the bile ducts. This accumulation leads to liver damage, scarring (cirrhosis), and eventually liver failure. Symptoms of biliary atresia usually appear within the first two months of life, and early diagnosis and treatment are crucial to prevent severe liver damage and complications. The primary treatment for biliary atresia is a surgical procedure called the Kasai procedure (hepatoportoenterostomy), which aims to restore bile flow from the liver to the small intestine. However, many children need to wait for several weeks to months for the Kasai procedure. During this waiting period, there is currently no drug that can be used to temporarily inhibit bile acid production in order to stop or slow the progression of the disease.

Chronic liver injury also disrupts bile acid homeostasis, leading to the accumulation of toxic bile acids in the liver [11]. This accumulation contributes to hepatocyte damage, inflammation, and oxidative stress, which in turn can stimulate hepatic stellate cells to transdifferentiate into myofibroblasts [12]. These activated myofibroblasts produce excessive amounts of extracellular matrix components, including collagen, resulting in the development of liver fibrosis and cirrhosis [13,14]. Serum levels of total bile acids increase with disease severity, which is highest in patients with cirrhosis [15,16]. Consequently, understanding the complex relationship between bile acid metabolism and liver fibrosis is critical for developing therapeutic strategies to prevent or treat liver fibrosis and associated liver diseases.

Sparked by the observation that DMSO suppresses genes implicated in bile acid synthesis within primary hepatocytes, an effect mirrored in mice, we embarked on our investigation. Progressing from this point, we assessed the capabilities of DMSO against bile-acid-induced liver damage, employing two distinctive mouse models. The first model utilized a 0.1% DDC diet to induce bile duct obstruction, simulating biliary atresia, while the second employed the CDA-HFD to trigger nonalcoholic steatohepatitis (NASH).

## 2. Materials and Methods

### 2.1. Mice

C57BL/6J mice were purchased from Jackson Laboratory. All animals were kept on a 12 h light–dark cycle in a temperature-controlled environment. Mice could freely access normal water or water containing 1–2% DMSO and were fed either a standard chow diet, a CDA-HFD diet (L-amino acid with 45% kcal fat with 0.1% methionine, no added choline, and 1% cholesterol; Research Diets, A16092003i, New Brunswick, NJ, USA), or a DDC diet (diethyl 1,4-dihydro-2,4,6-trimethyl-3,5-pyridinedicarboxylate, sc-239721, Santa Cruz, CA, USA). DMSO was administered by adding it to regular water at different concentrations for mice to take ad libitum, or via one bolus of gavage or a daily oral gavage at a dosing volume of 15 μL/g body weight following the standard gavage procedure. All animal procedures were approved by the Institutional Animal Care and Use Committee of Baylor College of Medicine (BCM).

### 2.2. Cell Culture

Primary mice hepatocytes were isolated in situ via liver perfusion, as previously described [17]. Briefly, mice aged 8–10 weeks were anesthetized via intraperitoneal injection of anesthesia mix, which contains ketamine (100 mg/kg body weight) and xylazine (10 mg/kg body weight). Then, blood was washed from the liver via perfusion (flow rate: 3 mL/min; 15 min; 37 °C) with a washing buffer (HBSS, no Ca^2+^, no Mg^2+^, no phenol red; 25 mM HEPES, 0.5 mM EDTA, pH 7.4). After removing the blood, the liver was further perfused (flow rate: 2 mL/min; 10 min; 37 °C) with the digestion buffer (HBSS with Ca^2+^, Mg^2+^, and phenol red; 25 mM HEPES, 25 μg/mL Liberase (Sigma Aldrich, 5401119001, St. Louis, MO, USA), pH 7.4). Then, the liver was dissected out gently, the gall bladder was removed, and the liver was placed in a 10 cm plate with a pre-chill digestion buffer without Liberase on ice. The liver sack was ruptured with fine-tip forceps in several locations along the liver surface to gently release cells into the buffer. Afterward, the suspension was filtered using a 70 μm cell strainer, centrifuged at 50× *g* for 2 min at 4 °C with low acceleration and low braking. The pellet was suspended in 15 mL 75% Percoll (VWR, 89428-524, Radnor, PA, USA) in PBS and centrifuged (200× *g*, 10 min, 4 °C). Finally, the cell pellet obtained was suspended in 10 mL Williams E Medium (Thermo Fisher Scientific, 12551032, Waltham, MA, USA) containing 10% fetal bovine serum (FBS, Thermo Fisher Scientific, 16140071, Waltham, MA, USA), 100 UI/mL penicillin, and 100 μg/mL streptomycin (Thermo Fisher Scientific, 10378–016, Waltham, MA, USA). Cell viability (≥90%) was estimated immediately, and cell density was adjusted to ≈1 × 106 viable cells/mL and plated on collagen-coated cell culture plates. Three hours after seeding, the medium was changed to a warm maintenance medium. The next day, cells were treated with different concentrations of DMSO (Sigma Aldrich, D2650-100ML, St. Louis, MO, USA) for 12 h to determine changes in expression of bile-acid-related regulatory genes due to qPCR.

### 2.3. RNA Extraction and qRT-PCR

Real-time quantitative reverse transcription PCR (qRT-PCR) was conducted as previously described [18,19]. Briefly, RNA was isolated from frozen tissues or culture cells through homogenization in TRIzol Reagent (Invitrogen, Waltham, MA, USA). A total of 1 μg of RNA was used to transcribe cDNA using a reverse transcription kit (Bio-Rad, Hercules, CA, USA). qRT-PCR primers were obtained from Harvard PrimerBank [20] and are listed in Table 1. The messenger RNA levels were calculated using the comparative threshold cycle (Ct) method, normalized to gene *Rps16*.

### 2.4. Bile Acid Extraction and Measurement

Cellular, liver, and serum bile acid were extracted and measured using the mouse total bile acids assay kit (Crystal Chem, 80471, Chicago, IL, USA), according to the manufacturer’s protocol.

### 2.5. Histology and Sirius Red Staining

Liver tissue was excised and fixed overnight in 10% PBS-buffered formalin and thereafter stored in 50% ethanol. Tissues were sectioned (5 μm), rehydrated, and stained at the digestive disease center at Texas Children’s Hospital. Microscopic images were taken on a ZEISS ZEN lite imaging system.

### 2.6. Total Liver Collagen Extraction and Measurement

Total liver collagen was extracted and measured with the total collagen assay kit (Abcam, ab222942, Cambridge, UK) using manufacturer-provided protocol.

### 2.7. Serum Alanine Aminotransferase (ALT) Activity Measurement

Serum was collected from mice by spinning clotted blood samples at 2000× *g* at 4 °C for 10 min in a refrigerated benchtop microcentrifuge and transferring the supernatant to a microcentrifuge tube. ALT activities were each determined using a colorimetric kit at the BCM mouse phenotype core.

### 2.8. Reverse-Phase Protein Array

The reverse-phase protein array (RPPA) method was employed, utilizing 234 antibodies to examine a range of cellular signaling pathways and target proteins that may be affected by 1% DMSO treatment in primary mouse hepatocytes. The procedure followed previously established protocols with slight adjustments [21]. In brief, cell homogenates were used to create protein lysates, employing a modified Tissue Protein Extraction Reagent (T-PER; Pierce) and a mixture of protease and phosphatase inhibitors (Roche Life Science, Penzberg, Germany). These lysates were subsequently diluted to a concentration of 0.5 mg/mL total protein in a sodium dodecyl sulfate sample buffer and denatured on the same day. Concurrently, different protein lysate controls for total and phosphoproteins were established, such as a blend of lysates from NCI-60 cell lines (diluted to 0.5 mg/mL), a mix of lysates from various mouse tissues (diluted to 0.5 mg/mL), and a CellMix control containing lysates from four cell lines (MDA-MB-415, T-47D, pervanadate-treated HeLa, and calyculin A-treated Jurkat cells), identified through extensive RPPA-based cell line screening. The CellMix control, which underwent eight serial dilutions (0.0078 to 1 mg/mL), functioned as both a positive control and a standard for assessing antibody and array process quality. An Aushon 2470 Arrayer (Aushon BioSystems, Billerica, MA, USA) with a 40-pin (185 µm) configuration was employed to deposit samples and control lysates onto nitrocellulose-coated slides (Grace Bio-Labs, Bend, OR, USA) in an array format that accommodated 960 lysates/slide (2880 spots/slide). The slides were then subjected to validated antibodies targeting total and phosphoprotein proteins using an Autolink 48 automated slide stainer (Dako). Each slide was treated with a specific primary antibody, while a negative control slide was exposed to antibody diluent in place of the primary antibody during each staining run, which each consisting of approximately 30 slides. Biotinylated secondary antibodies were used to detect primary antibody binding, followed by the application of streptavidin-conjugated IRDye680 fluorophore (LI-COR Biosciences, Lincoln, NE, USA). The total protein content of each spotted lysate was assessed through fluorescent staining using Sypro Ruby Protein Blot Stain, following the manufacturer’s guidelines (Molecular Probes, Eugene, OR, USA).

### 2.9. Statistical Analysis

Results are shown as mean ± SEM. For experiments with only two groups, the Student’s *t*-test was utilized. For studies with three or more groups, one-way ANOVA was used, and for experiments with several groups with a balanced distribution of two factors, a two-way ANOVA test was used. Tukey’s test was used for post hoc analysis of comparisons within subgroups. RPPA data consisted of three technical replicates for each sample; the mean value of the triplicates for each sample and antibody was used for further analysis, and *p* values < 0.05 were considered statistically significant.

## 3. Results

**Figure 1:**
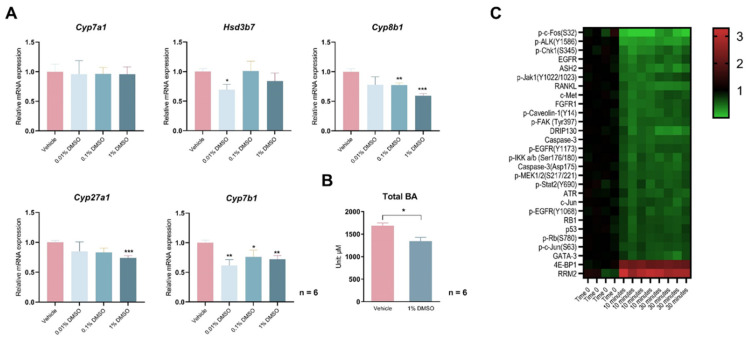
DMSO acutely inhibits bile acid (BA) synthase gene expression and reduces cellular BAs. (**A**) Expression of *Cyp7a1*, *Hsd3b7*, *Cyp8b1*, *Cyp27a1*, and *Cyp7b1* in primary hepatocytes treated with 0.01%, 0.1%, and 1% (*v*/*v*) DMSO for 12 h. (**B**) Total BA levels in hepatocytes treated with 1% DMSO for 24 h. For panels (**A**,**B**), *n* = 6 for each group. (**C**) Heatmap of changes in cellular signaling pathways detected by a reverse-phase protein array (RPPA) in primary hepatocytes treated with 1% (*v*/*v*) DMSO for 10 min or 30 min. Protein intensities are displayed as colors ranging from green to red as shown in the key. All data are mean ± SEM; * *p* < 0.05, ** *p* < 0.01, *** *p* < 0.001, relative to the control group.

Treating freshly isolated and attached hepatocytes with different concentrations of DMSO for 12 h significantly reduced the expression of *Cyp8b1* in the canonical bile acid synthesis pathway and of *Cyp27a1* and *Cyp7b1* in the non-classical synthesis pathway (Figure 1A). As the rate-limiting enzyme *Cyp7a1* in the classical pathway was not affected, those hepatocytes displayed a mild but statistically significant 20.3% reduction in total cellular bile acid contents (Figure 1B). Reasoning that DMSO may alter cellular signaling to change bile acid synthesis, we performed a reverse-phase protein array (RPPA) assay, utilizing 234 antibodies, to examine a range of cellular signaling pathways and target proteins that may be affected by 1% (*v*/*v*) DMSO treatment in primary mouse hepatocytes. Of them, signals from 214 antibodies were validated and used for statistical analysis (Supplementary Table S1). After filtering the results with fold change > 50% (*p* < 0.01) for both 10 min vs. 0 min and 30 min vs. 0 min comparisons, 28 antibody-detected targets were left. Of them, only eukaryotic initiation factor 4E-binding protein 1 (4E-BP1) and ribonucleotide reductase regulatory subunit M2 (RRM2) were significantly upregulated by DMSO, with other targets all acutely suppressed by DMSO treatment, which includes phosphor-c-Fos (S32), with a 98% reduction after 10 min of DMSO treatment (Figure 1C).

**Figure 2:**
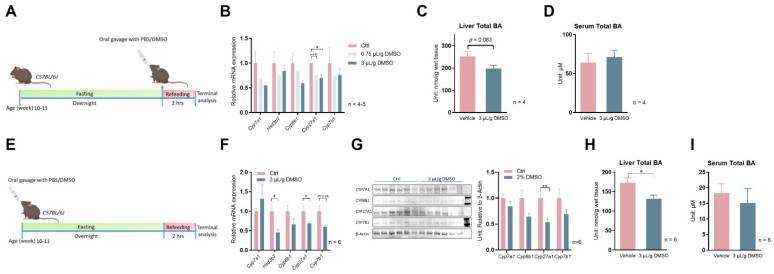
DMSO inhibits expression of bile acid synthesis in mouse livers. (**A**) Schematic representation of mouse treatment for panels (**B**–**D**). (**B**) Expression of *Cyp7a1*, *Hsd3b7*, *Cyp8b1*, *Cyp27a1*, and *Cyp7b1* after one bolus of 0.75 or 3 μL/g (*v*/*w*) DMSO oral gavage (15 μL/g body weight) and 2 h refeeding following overnight fasting in C57Bl/6J wild–type (WT) mice; *n* = 5 for the control group, *n* = 5 for the 0.75 μL/g DMSO group, *n* = 4 for the 3 μL/g DMSO group. (**C**) Total liver BA level, (**D**) serum total BA level 2 h after one bolus of 3 μL/g (*v*/*w*) DMSO oral gavage (15 μL diluted DMSO/gram body weight) in WT mice. For panels (**C**,**D**), *n* = 4 for each group. (**E**) Schematic representation of mouse treatment for panels (**F**–**I**). (**F**) Expression of *Cyp7a1*, *Hsd3b7*, *Cyp8b1*, *Cyp27a1*, and *Cyp7b1* 24 h after one bolus of 3 μL/g (*v*/*w*) DMSO oral gavage (15 μL diluted DMSO/gram body weight) in WT mice. (**G**) Western blots of CYP7A1, CYP8B1, CYP27A1, CYP7B1 proteins; densitometry results of proteins signal normalized to β–Actin are shown on the right (Full Western blots figures refer to Supplementary Materials). (**H**) Total liver BA level, (**I**) serum total BA level from panel (**E**); for panel (**F**–**I**), *n* = 6 for each group. All data are mean ± SEM; * *p* < 0.05, ** *p* < 0.01, relative to the control group.

Encouraged by these positive data, we decided to determine whether DMSO administration to mice would suppress bile acid synthesis gene expression in the liver. We first administered 5% or 20% DMSO dissolved in water to C57Bl/6J wild-type mice at 15 µL/g body weight, which equals 0.75 µL/g or 3 µL/g body weight, in the morning after overnight fasting. Two hours after refeeding, hepatic expression of bile acid synthesis genes was evaluated (Figure 2A). *Cyp27a1* was significantly suppressed by the higher dose of DMSO treatment, while *Cyp7a1* and *Cyp8b1* also showed a trend of decreasing expression but without statistical significance (Figure 2B). The inhibition of bile acid synthesis genes, translating to a mild 21.4% reduction in total liver bile acids (*p* = 0.083), while serum total bile acids were not affected (Figure 2C,D). When we extended the treatment duration to 24 h (Figure 2E), the higher dose of DMSO treatment (3 µL/g) still significantly repressed *Cyp27a1*, with additional inhibition of *Hsd3b7* and *Cyp7b1*, suggesting the slow kinetics in inhibition of those genes by DMSO in mice (Figure 2F). The inhibition of mRNA translates to significantly reduced CYP27A1 protein levels in liver lysates revealed by Western blots (Figure 2G) and a 23.9% reduction (*p* = 0.023) in liver total bile acids (Figure 2H). However, serum total bile acid levels were still not affected by DMSO (Figure 2I).

**Figure 3:**
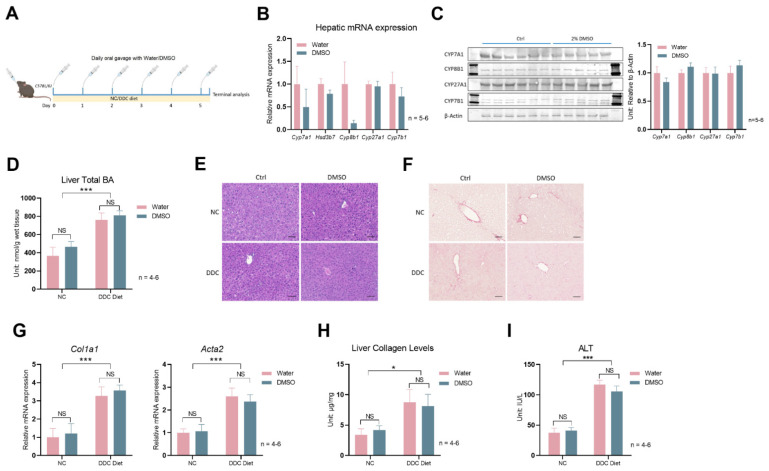
DMSO does not protect mice from 0.1% DDC–diet–induced liver damage. (**A**) Schematic representation of mouse treatment: C57Bl/6J WT mice were kept on normal chow (NC) or 0.1% DDC diet with a daily oral gavage of PBS or 3μL/g body weight (*v*/*w*) DMSO (15 μL/g body weight) for 5 days. (**B**) Expression of *Cyp7a1*, *Hsd3b7*, *Cyp8b1*, *Cyp27a1*, and *Cyp7b1* in liver. (**C**) Western blots of CYP7A1, CYP8B1, CYP27A1, CYP7B1 proteins; densitometry results of proteins signal normalized to β–Actin are shown on the right (Full Western blots figures refer to Supplementary Materials). For panel (**B**,**C**), *n* = 6 for the ctrl group, *n* = 5 for the 3μL/g DMSO group. (**D**) Total liver BA level in WT mice kept on NC or 0.1% DDC diet with regular water or DMSO water treatment for 5 days. (**E**) Representative liver H&E staining and (**F**) Sirius Red staining in mice described in panel (**D**). (**G**) Hepatic expression of *Col1a1* and *Acta2* in mice from panel (**D**). (**H**) Liver collagen levels in mice from panel (**D**). (**I**) Serum alanine transaminase (ALT) levels in mice from panel (**D**). For panels (**D**–**I**), *n* = 4 for the control group, *n* = 5 for the DMSO group, *n* = 6 for the DDC group, and *n* = 5 for the DDC + DMSO group. Two–way ANOVA was used for panels (**D**), (**G**–**I**). All data are mean ± SEM. NS: not significant; * *p* < 0.05, *** *p* < 0.001.

Next, we sought to test whether the inhibition of bile acid synthesis by DMSO may alleviate bile-acid-induced liver damage. We first chose a mouse model of DDC diet (diethyl 1,4-dihydro-2,4,6-trimethyl-3,5-pyridinedicarboxylate, 0.1%)-induced bile duct obstruction and acute accumulation of bile acids. Mice treated with 0.1% DDC became sick rapidly, and the experiment was terminated 5 days after mice were switched to a 0.1% DDC diet (Figure 3A). Daily DMSO gavage reduced the mean of hepatic *Cyp8b1* expression but did not reach statistical significance (*p* = 0.14), largely due to the very high intragroup variation (Figure 3B). The protein levels of bile acid synthesis enzymes were not different between the vehicle and DMSO treatment mice on 0.1% DDC diet (Figure 3C). The 0.1% DDC diet significantly increased hepatic bile acid levels, but DMSO had no effect on hepatic total bile acid levels (Figure 3D). Liver histology revealed by hematoxylin and eosin (H&E) staining remains similar between vehicle (control) and DMSO treatment mice; DDC diet did not even significantly affect liver histology despite the mice being sick (Figure 3E). Sirius Red staining indicates higher liver fibrosis levels in 0.1% DDC diet treatment mice, but DMSO had no effect (Figure 3F). Hepatic expression of *Col1a1* and *Acta2* were significantly increased by the 0.1% DDC diet treatment, agreeing with the Sirius Red staining result of increased liver fibrosis; DMSO had no effect on the expression of the two genes (Figure 3G). Liver collagen levels, another indicator of liver fibrosis levels, showed a similar pattern (Figure 3H). Serum ALT levels were increased by the 0.1% DDC diet, and DMSO had no effect (Figure 3I). Altogether, DMSO could not reduce bile acid accumulation, nor reduce the liver damage resulting from a 0.1% DDC diet treatment.

**Figure 4:**
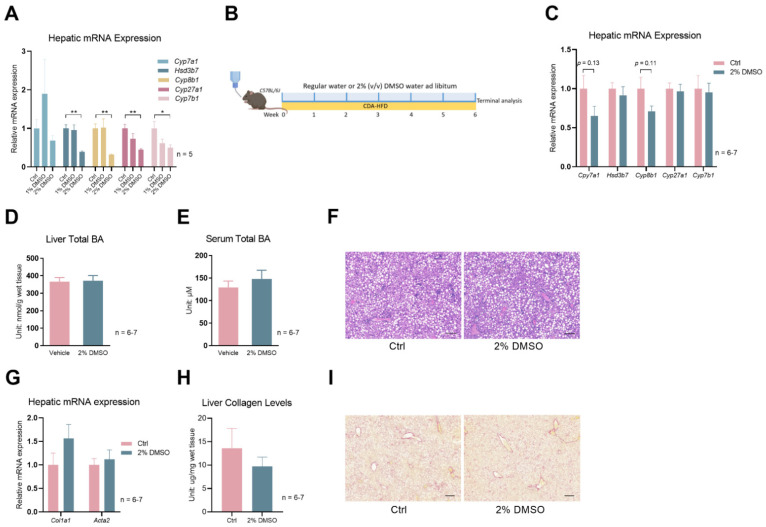
DMSO does not protect mice from CDA–HFD-induced liver fibrosis. (**A**) Expression of *Cyp7a1*, *Hsd3b7, Cyp8b1, Cyp27a1*, and *Cyp7b1* in WT mice kept on 1% or 2% (*v*/*v*) DMSO water for one week; *n* = 5 for each group. (**B**) Schematic of mouse treatment for panels (**C**–**I**): C57Bl/6J WT mice were all treated with CDA–HFD, but with or without 2% (*v*/*v*) DMSO in their drinking water. The treatment lasted for 6 weeks. (**C**) Hepatic expression of *Cyp7a1*, *Hsd3b7*, *Cyp8b1*, *Cyp27a1*, and *Cyp7b1* in mice described in Panel (**B**). (**D**) Total BA levels in the liver. (**E**) Total BA levels of serum. (**F**) Representative H&E staining of liver sections from mice described in panel (**B**). Scale bar: 200 µm. (**G**) Hepatic expression of *Col1a1* and *Acta2*. (**H**) Liver collagen levels in the mice described in panel (**B**). (**I**) Representative Sirius Red staining of liver sections from mice described in panel (**B**). Scale bar: 200 µm. For panels (**C**–**I**), *n* = 7 for the control group and *n* = 6 for the 2% DMSO group. All data are mean ± SEM; * *p* < 0.05, ** *p* < 0.01, relative to the control group.

Lastly, given the rapid development of acute hepatic and systemic failure in the 0.1% DDC-diet mouse model, we employed another mouse model: CDA-HFD (choline-deficient, L-amino acid-defined, high-fat diet). Feeding mice a CDA-HFD is a widely used method of inducing liver fibrosis in as short as 6–9 weeks [22,23]. Due to the nature of the long-term treatment of CDA-HFD-induced liver fibrosis, we first tested a new approach other than the use of oral gavage to administer DMSO to mice. Adding DMSO to drinking water (2% *v*/*v*) was sufficient to suppress several bile acid synthesis genes to a similar degree of DMSO oral gavage (Figure 4A). Then, we treated a cohort of mice with CDA-HFD and with or without DMSO in their drinking water (Figure 4B). Gene expression analysis shows mild trends of reductions in hepatic *Cyp7a1* and *Cyp8b1* levels in the DMSO treatment group for mice treated with CDA-HFD for 6 weeks (Figure 4C). However, total liver bile acid and serum bile acid levels were not reduced by DMSO (Figure 4D,E), as seen in the DDC diet treatment experiment. CDA-HFD treatment leads to the accumulation of lipid droplets and the infiltration of inflammatory monocytes in the liver, but DMSO did not reduce the severity of those two processes (Figure 4F). Liver fibrosis levels, indicated by *Col1a1* and *Acta2* expression, and hepatic collagen levels were not different (Figure 4G,H). Sirius Red staining, which stains collagen fibers red, showed no difference in intensity between water- and DMSO-treated mice livers (Figure 4I).

## 4. Discussion

As bile acid homeostasis plays a vital role in the digestion and absorption of dietary fats, along with the elimination of cholesterol and other waste products from the liver [4], alteration of this homeostasis contributes to the dysfunction of systemic metabolism, as well as several forms of liver disease [24]. One extreme example is biliary atresia in infants [10]. When the bile acid builds up in the liver of those patients, finding a treatment to reduce bile acid accumulation before a Kasai procedure could save many lives.

Inhibiting liver bile acid synthesis is postulated to be one way to reduce bile acid accumulation in the liver. Obeticholic acid (Ocaliva) is the only FDA-approved drug that inhibits bile acid synthesis. It works through activating farnesoid X receptor (FXR) in the liver and intestine to reduce the production of bile acids in the liver. While it has been approved to treat primary biliary cholangitis (PBC), a chronic liver disease caused by damage to the bile ducts in the liver, its safety profile is poor, casting doubt on whether it should be used with infants.

Prompted by a serendipitous observation in primary hepatocytes that DMSO can inhibit several genes in the bile acid synthesis pathway, we found this treatment effect translates into mice with administration of a single dose or orally administered DMSO to reduce postprandial bile acid synthesis gene expression and hepatic total bile acid levels. A relatively mild reduction in total bile acid levels after DMSO treatment might stem from the inhibition of alternative pathway enzyme CYP27A1 instead of the rate-limiting enzyme CYP7A1 in the classical pathway by DMSO, as the alternative bile acid synthesis pathway only contributes to about 10% of total liver bile acid synthesis. This mild reduction may be masked when mice are not in a uniform feeding state, which contributes to the variations in hepatic total bile acid levels, as seen in the DDC diet study. Of note, a previous report suggested that 9-day 2% (*v*/*v* in tap water) DMSO treatment directly inhibits the activity of the hepatic rate-limiting enzyme CYP7A1 (control: 9.7 +/− 1.0 (*n* = 6) vs. DMSO: 4.3 +/− 0.7) in rats [25]. However, the hepatic total bile acid levels and mechanism of CPY7A1 inhibition by DMSO were not determined. We only saw a trend in the reduction in *Cyp7a1* expression, but for the most part, it was not statistically significant.

When mice are fed with DDC, it leads to the formation of porphyrin plugs within the small bile ducts, which obstruct the flow of bile, mimicking symptoms of cholestasis in humans. The resulting bile duct injury and the body’s response to it lead to fibrosis and the formation of new bile ducts. The progression of cholangiopathies usually takes several weeks in this mouse model [26,27]; however, in our hands, the symptom quickly progressed to liver failure and death of the animals. We saw an approximately onefold increase in total liver bile acid levels and more than a onefold increase in liver fibrosis levels, gauged by *Col1a1* expression and total liver collagen levels. Serum ALT levels were also significantly elevated by the DDC treatment. Despite having the effect of inhibiting bile acid synthesis genes via oral administration of DMSO, daily DMSO treatment failed to reduce liver or serum bile acid levels and could not provide any hepatic protection based on the liver collagen and serum ALT data.

In the second mouse model of CDA-HFD treatment, DMSO treatment in drinking water delivered a similar degree of suppression of the bile acid synthesis genes in healthy animals and showed a trend of suppressing hepatic *Cyp7a1* and *Cyp8b1* expression in mice treated with 6 weeks of CDA-HFD. However, total liver bile acid levels and liver fibrosis levels were not improved by DMSO water treatment for mice treated with CDA-HFD.

DMSO’s predominant effect is the inhibition of *Cyp27a1*, seen in primary hepatocytes 2 h and 24 h after orally administering or continuously treating mice with DMSO water. However, the suppression is absent in mice treated with a DDC diet or CDA-HFD, suggesting loss of the regulation of *Cyp27a1* by DMSO under those pathological conditions.

Hepatic bile acid levels are regulated through a complex network of pathways and feedback mechanisms that maintain a delicate balance between bile acid synthesis, transport, and metabolism [24]. One explanation of the very moderate or absence of reduction in hepatic bile acids that we witnessed in this study were probably complicated by the export from the hepatocytes, which may be a more dominant determining factor in the hepatic bile acid homeostasis.

Last but not least, our data add additional evidence that DMSO is bioactive. However, it is also important to note that the effects of DMSO on hepatocytes are concentration-dependent and can vary based on experimental conditions. The exact mechanisms underlying DMSO’s bioactivity in hepatocytes remain an active area of investigation. Our RPPA also provides valuable information that aids in understanding DMSO’s bioactivity, at least at the cellular level. In as little as 10 min, the DMSO dramatically altered protein abundance and phosphorylation of many kinases and receptors. Among those changed molecules, ERK and JNK activation has been implicated in regulating *Cyp7a1* levels [28], and c-fos is implicated in regulating ASBT promoter activity in CT-26 cells [29]. However, whether they are responsible for bile acid synthesis gene changes mediated by DMSO treatment requires further study.

## 5. Limitations

We acknowledge certain limitations of this study. First, the animal experiments were conducted under the guiding principle of the 3Rs: reduce, reuse, and recycle. A strategy of reduction led to small sample sizes for some experiments, potentially affecting our ability to discern statistically significant differences for certain parameters. Nonetheless, these samples provided adequate information with which to address the research question. Second, during the study, a few mice receiving 0.1% DDC treatment were euthanized when their condition appeared morbid, potentially leading to substantial variations in bile acid synthesis gene expression within the group. This variation could have obscured statistical significance, despite notable differences in mean values.

Furthermore, we recognize that the prospect of employing DMSO for treating liver fibrosis in a clinical setting is slim, notwithstanding some studies suggesting its anti-fibrotic potential and hepatoprotection in small animal models [30,31,32,33,34]. This limitation primarily stems from a lack of efficacy or just showing mediocre effectiveness in animal studies and the challenges associated with conducting a double-blinded clinical trial using DMSO, compounded by a lack of economic incentives to initiate such trials.

## 6. Conclusions

Contrary to the canonical view that DMSO is an inert chemical, it effectively reduces bile acid content in primary hepatocytes and in the livers of healthy mice post-refeeding, achieved through the suppression of several synthesis enzymes’ transcription and reduction of the CP27A1 protein. However, in two mouse models of hepatic bile acids’ dyshomeostasis, it failed to confer any discernible benefits, potentially due to the loss of its inhibitory effect on bile acid production under those pathological conditions.

## Figures and Tables

**Table 1:** List of primers used in this study.

Name of the Gene	PCR Product Length	Name of the Primer	Sequence (5′ to 3′)
*Cyp7a1*	100 bp	Forward	GGGATTGCTGTGGTAGTGAGC
		Reverse	GGTATGGAATCAACCCGTTGTC
*Hsd3b7*	197 bp	Forward	GGGAGCTGCGTGTCTTTGA
		Reverse	GTGGATGGTCTTTGGACTGGC
*Cyp8b1*	112 bp	Forward	CCTCTGGACAAGGGTTTTGTG
		Reverse	GCACCGTGAAGACATCCCC
*Cyp27a1*	139 bp	Forward	CCAGGCACAGGAGAGTACG
		Reverse	GGGCAAGTGCAGCACATAG
*Cyp7b1*	160 bp	Forward	GGAGCCACGACCCTAGATG
		Reverse	TGCCAAGATAAGGAAGCCAAC
*Col1a1*	137 bp	Forward	GATGGATTCCCGTTCGAGTA
		Reverse	ATGTAGGCTACGCTGTTCTT
*Acta2*	102 bp	Forward	GTCCCAGACATCAGGGAGTAA
		Reverse	TCGGATACTTCAGCGTCAGGA
*Rps16*	127 bp	Forward	CACTGCAAACGGGGAAATGG
		Reverse	CACCAGCAAATCGCTCCTTG

## Data Availability

All data supporting the findings of this study are available upon reasonable request. Mouse models are available from the corresponding author on request.

## Supplementary Materials

The following are available online at https://www.mdpi.com/article/10.3390/biology12081105/s1. Figure S1: Full western blot figures. Table S1: The reverse-phase protein array raw and analyzed data.
