# Supplementary material for: Dimethyl Sulfoxide Inhibits Bile Acid Synthesis in Healthy Mice but Does Not Protect Mice from Bile-Acid-Induced Liver Damage
Source: Biology (Basel). 2023 Aug 9;12(8):1105. doi: 10.3390/biology12081105 (PMC10452260; doi:10.3390/biology12081105)
Supplement: Supplementary file 1 [file biology-12-01105-s001.zip › biology-2428311-Figure S1.pdf]

Biology-2428311 Full WB figures

Dimethyl sulfoxide inhibits bile acid synthesis in healthy mice but does not protect mice from bile acid-induced liver damage

Fig. 2G

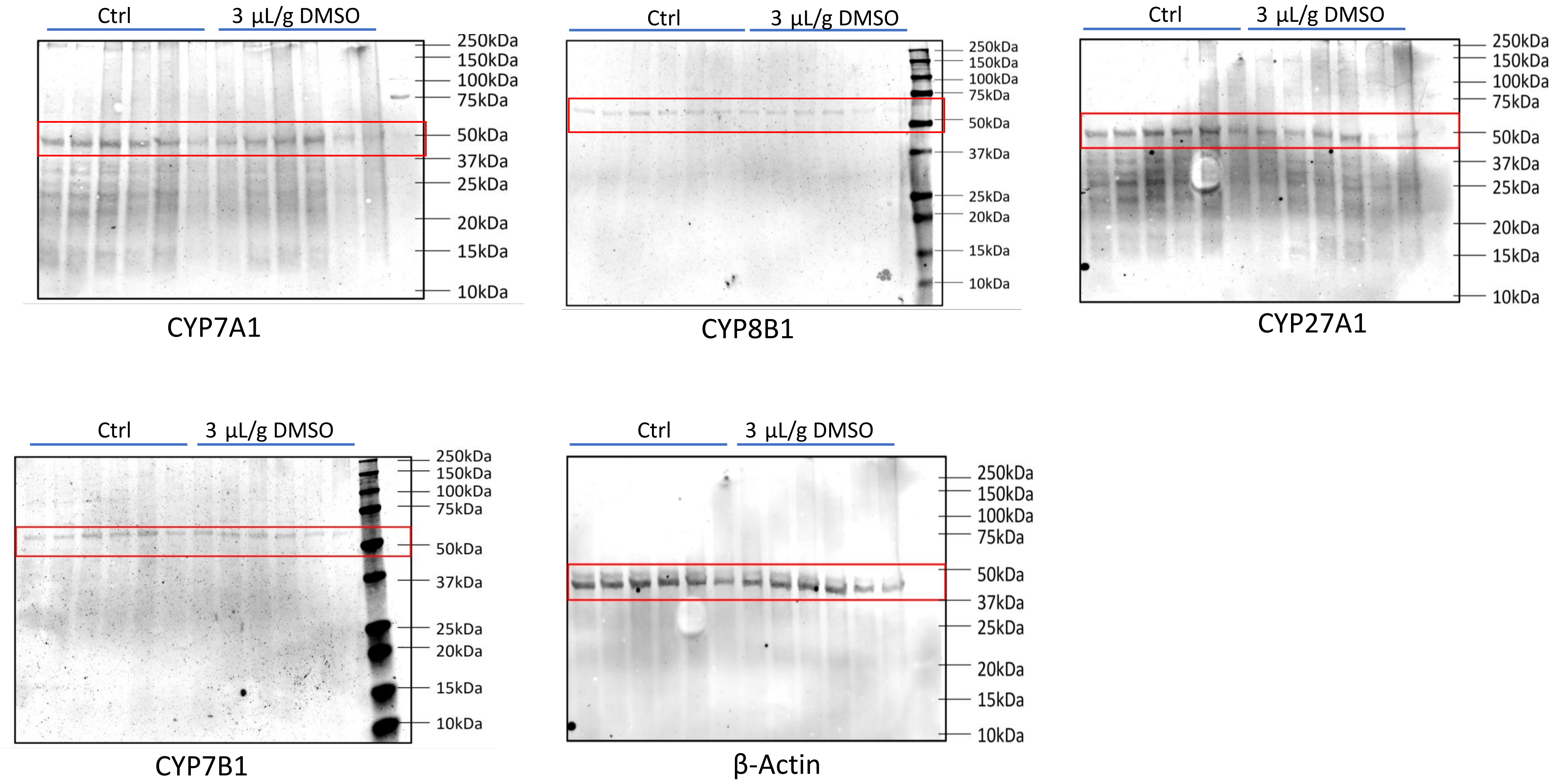
Ctrl
3 µL/g DMSO
250kDa
150kDa
100kDa
75kDa
50kDa
37kDa
25kDa
20kDa
15kDa
10kDa
CYP7A1
Ctrl
3 µL/g DMSO
250kDa
150kDa
100kDa
75kDa
50kDa
37kDa
25kDa
20kDa
15kDa
10kDa
CYP8B1
Ctrl
3 µL/g DMSO
250kDa
150kDa
100kDa
75kDa
50kDa
37kDa
25kDa
20kDa
15kDa
10kDa
CYP27A1
Ctrl
3 µL/g DMSO
250kDa
150kDa
100kDa
75kDa
50kDa
37kDa
25kDa
20kDa
15kDa
10kDa
CYP7B1
Ctrl
3 µL/g DMSO
250kDa
150kDa
100kDa
75kDa
50kDa
37kDa
25kDa
20kDa
15kDa
10kDa
β-Actin

Fig. 3C

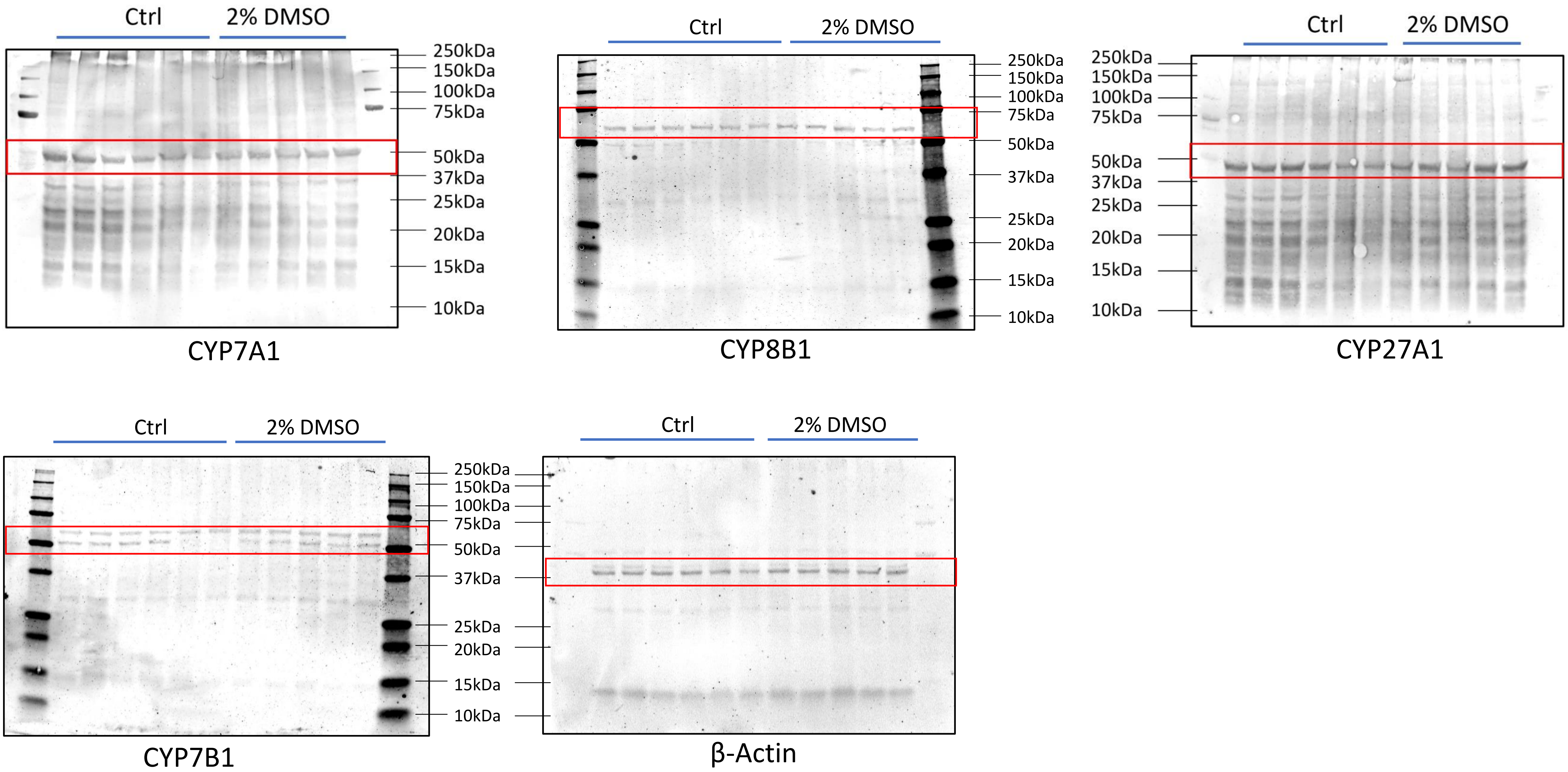
Ctrl
2% DMSO
250kDa
150kDa
100kDa
75kDa
50kDa
37kDa
25kDa
20kDa
15kDa
10kDa
CYP7A1
CYP8B1
CYP27A1
CYP7B1
β-Actin
